# Combination of atorvastatin and ticagrelor associated with rhabdomyolysis: A case report

**DOI:** 10.1097/MD.0000000000045189

**Published:** 2025-10-10

**Authors:** Bin Li, Ling Zhao

**Affiliations:** a Department of Pharmacy, The People’s Hospital Medical Group of Xiangzhou, Zhuhai, Guangdong, China; b Department of Intensive Care Unit, The People’s Hospital Medical Group of Xiangzhou, Zhuhai, Guangdong, China.

**Keywords:** acute coronary syndrome, drug-drug interactions, rhabdomyolysis, statins, ticagrelor

## Abstract

**Rationale:**

Statin-associated myalgia are common, but rhabdomyolysis remains a rare and severe adverse effect. There are scant reports, both domestically and internationally, on rhabdomyolysis induced by the combination of atorvastatin and ticagrelor.

**Patient Concerns:**

A 57-year-old male patient with acute coronary syndrome underwent emergency percutaneous coronary intervention surgery. He was prescribed ticagrelor (90 mg twice daily) and atorvastatin (40 mg once daily). Three days after treatment initiation, his creatine kinase, creatinine, and myoglobin levels increased markedly, and he developed acute renal failure.

**Diagnoses:**

Rhabdomyolysis was diagnosed based on the medication history, significantly elevated creatine kinase and myoglobin levels, and the onset of acute renal failure.

**Interventions:**

Management included immediate drug discontinuation, aggressive fluid resuscitation, continuous renal replacement therapy, and extracorporeal membrane oxygenation.

**Outcomes:**

Despite these efforts, the patient died.

**Lessons:**

This case aims to alert clinicians to the potential for this rare but fatal adverse effect, particularly in high-risk patients receiving aggressive statin therapy. It also discusses the rationale for considering alternative lipid-lowering strategies in acute coronary syndrome patients with multiple risk factors for statin-induced myotoxicity to mitigate the risk of rhabdomyolysis.

## 1. Introduction

Current guidelines strongly recommend ticagrelor and high-intensity statins as first-line therapies for patients with acute coronary syndrome (ACS).^[1]^ Common adverse reactions to statins include myalgia, but rhabdomyolysis (RM) is a rare and most severe adverse reaction, with an incidence rate of <0.1%.^[2]^ It is well established that drug-drug interactions (DDIs) and high-intensity statins are both significant risk factors. The risk of statin-induced myopathy is significantly increased by concomitant use of drugs that inhibit the cytochrome P450 3A4 isoenzyme, which is responsible for the metabolism of several statins, including atorvastatin and simvastatin. Well-known perpetrators of such interactions include posaconazole, verapamil, clarithromycin, and ritonavir. Emerging evidence suggests a potential DDIs between atorvastatin and ticagrelor, involving multiple mechanisms including CYP3A4 and P-glycoprotein. However, there are exceedingly few reported cases, both domestically and internationally, of RM directly attributed to the combination of these 2 drugs. This report analyzes a case of ACS in which a patient developed RM and subsequent acute renal failure (ARF) following the co-administration of ticagrelor and a high-intensity statin. This case serves to highlight the potential for rare but serious adverse effects of statins, particularly in the context of DDIs and other patient-specific risk factors. Furthermore, it underscores the critical need for clinicians to be aware of these risks and to consider alternative treatment strategies for ACS patients at high risk for statin-related myotoxicity.

## 2. Case presentation

**Figure 1.**
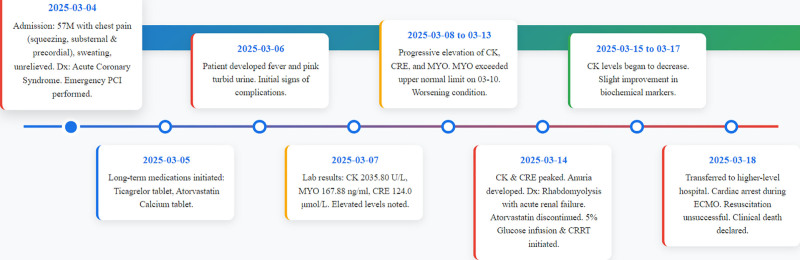
This case report adheres to the CARE reporting guidelines for the presentation of a case study. Historical and current information from this episode of the case is organized as a timeline. CK = creatine kinase, CRE = creatinine, CRRT = continuous renal replacement therapy, Dx = diagnosis, ECMO = extracorporeal membrane oxygenation, Lab = laboratory examinations, MYO = myoglobin, PCI = percutaneous coronary intervention.

**Figure 2.**
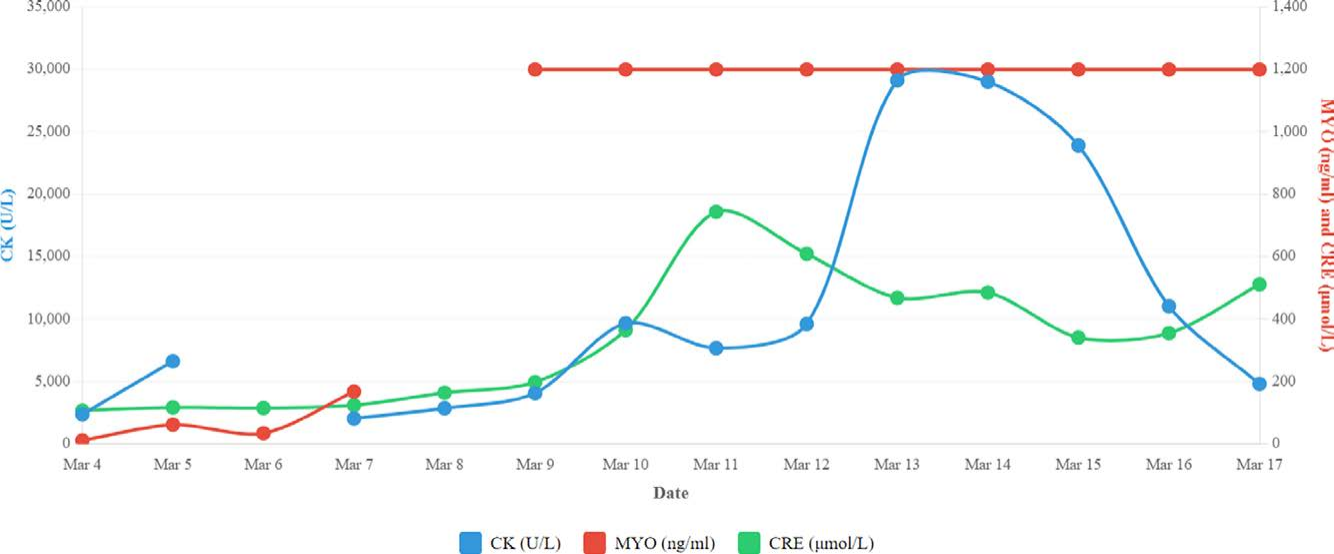
Changes in CK, MYO, and CR levels during the patient’s hospitalization. CK = creatine kinase, CRE = creatinine, MYO = myoglobin.

On March 4, 2025, a 57-year-old male patient (weight, 60 kg; height, 170 cm; body mass index, 20.76 kg/m²) presented with chest tightness and pain without obvious precipitating factors, lasting for 1 hour and fifty minutes. The pain was predominantly located in the precordial and retrosternal regions, characterized as a persistent, pressure-like or crushing sensation, and was associated with profuse sweating. It was not relieved by rest or change in position. The initial ECG at the chest pain center demonstrated sinus rhythm with ST-segment depressions, as documented in the physician’s admission note. However, the original ECG tracing is no longer retrievable due to the hospital’s record retention policy beyond a 6-month period. The clinical team at the time interpreted this finding as acute myocardial ischemia. The patient had a smoking history but no other significant medical history. C-reactive protein level 10.09 ng/mL; myoglobin (MYO) 11.4 ng/mL; creatine kinase (CK) 2370.60 U/L; creatinine (CRE) 107.0 μmol/L; alanine aminotransferase 55.07 U/L; aspartate transaminase 176.0 U/L. Diagnosis: “ACS.” Immediately administered aspirin 300 mg and clopidogrel 600 mg orally, the patient initially refused invasive angiography. However, during observation, the patient’s condition rapidly deteriorated with the onset of acute pulmonary edema, manifested by coughing of pink frothy sputum. This life-threatening complication warranted immediate revascularization. After reiterated communication and consent, the patient was urgently transferred to the catheterization lab for emergency PCI. March 5, 2025: CK 6624.80 U/L; cardiac troponin I (cTnI) 81.99 ng/mL; MYO 61.81 ng/mL; CRE 117.0 μmol/L; thyroid-stimulating hormone 2.007 μIU/mL. Long-term prescription: Aspirin 100 mg once daily orally, ticagrelor 90 mg twice daily orally, atorvastatin 40 mg once daily orally, ezetimibe 10 mg once daily orally, pantoprazole sodium injection for acid suppression and gastric protection. Administered: Remifentanil injection for analgesia, midazolam injection for sedation, norepinephrine injection for blood pressure elevation, dopamine injection for cardiac stimulation, furosemide injection for diuresis. March 6, 2025: Patient developed fever, pink cloudy urine (suggestive of myoglobinuria). CK not tested, cTnI > 50 ng/mL; MYO 34.3 ng/mL; CRE 115.0 μmol/L. Suspected aspiration pneumonia, long-term administration of piperacillin sodium and tazobactam injection. March 7, 2025: CK 2035.80 U/L; cTnI >100 ng/mL; MYO 167.88 ng/mL; CRE 124.0 μmol/L; alanine aminotransferase 131.29 U/L; aspartate transaminase 490.8 U/L; Liver function markers were elevated compared with prior levels. March 8, 2025, to March 14, 2025: CK, CRE, and aspartate transaminase levels gradually increased, reaching peak values on March 13, 2025 (29,125 U/L, 744.0 μmol/L, and 701.7 U/L) (Fig. 1).^[3]^ On March 10, 2025, the patient’s MYO level exceeded 1200 ng/mL, surpassing the upper reference limit, and he developed anuria. A diagnosis of RM complicated by ARF and heart failure was considered. Atorvastatin was immediately discontinued. Fluid therapy with 5% glucose injection was initiated, and continuous renal replacement therapy was commenced. From March 15 to March 17, 2025, a slight decrease in CK levels was observed (Fig. 2). However, on March 18, 2025, the patient’s condition deteriorated acutely. His heart rate dropped to 38 beats per minute, and he became hypotensive and unresponsive to verbal stimuli. Immediate external cardiac compression was performed. Although spontaneous circulation was successfully restored initially, the patient was transferred to a higher-level hospital for further management. During subsequent preparation for extracorporeal membrane oxygenation (ECMO), the patient suffered a cardiac arrest. Resuscitation efforts were unsuccessful, and the patient was pronounced clinically dead.

## 3. Diagnostics assessment

RM is clinically defined as a CK level exceeding 40 times the upper limit of normal (ULN), accompanied by myoglobinuria and/or ARF.^[4]^ At our institution, the ULN for CK is 170 U/L and for MYO is 58 ng/mL. The patient presented with a CK level of 29,125 U/L (~171 times ULN), MYO > 1200 ng/mL, CRE 744 μmol/L, and ARF, fulfilling the clinical diagnostic criteria for RM.

## 4. Discussion

The risk factors for atorvastatin-associated RM in this patient include the following: Statin type: Certain statins including lovastatin, simvastatin, and atorvastatin, are extensively metabolized by the cytochrome P450 3A4 enzyme. This significantly increases the risk of muscle injury.^[5]^ Dose: A clear dose–response relationship exists for statin-induced myopathy, including RM. Analyses of adverse event reports from Canada and the United States specific to atorvastatin showed that, compared with a 10 mg daily dose, the risk of RM was significantly higher at 40 mg (OR 3.8; 95% CI, 2.3–6.6; *P* < .0001) and 80 mg (OR 11.3; 95% CI, 6.4–20.4; *P* < .0001).^[6]^ Race: Pharmacokinetic studies indicate that plasma concentrations of atorvastatin and its active metabolites are approximately twofold higher in East Asian subjects compared to Caucasians. This is primarily attributed to differences in the prevalence of variant alleles encoding statin-metabolizing enzymes and membrane transporters. Furthermore, a genome-wide association study identified that common variants in the SLCO1B1 gene significantly alter the risk of statin-induced myopathy. This gene encodes the organic anion-transporting polypeptide 1B1, which is responsible for the hepatic uptake of most statins.^[7]^ Baseline CK levels. DDIs: Atorvastatin and ticagrelor have a clinically significant DDIs, with a Lexicomp risk rating of Category C (monitor therapy).^[8]^ The potential mechanisms involve cytochrome P450 3A4, organic anion transporters, P-glycoprotein, and glucuronidation. Studies indicate that ticagrelor increases systemic exposure to atorvastatin and its metabolites: atorvastatin by 36%, atorvastatin lactone by 32%, 2-hydroxyatorvastatin by 33%, and 4-hydroxyatorvastatin by 67%.^[9]^ This is clinically relevant since both the inactive lactone form and hydroxy metabolites may contribute to myopathy.^[10]^ Epidemiological evidence from a large drug safety database^[11]^ supports this risk: among 9489 reports of statin-associated RM out of 11,431,708 adverse reaction reports, 2464 cases (26%) occurred in patients concurrently receiving antiplatelet therapy. Specifically, co-administration of ticagrelor with atorvastatin (ROR 1.30; 95% CI, 1.02–1.65) or rosuvastatin (ROR 1.90; 95% CI, 1.42–2.54) was associated with a statistically significant increase in RM reporting compared to statin use alone.

During hospitalization, the concurrent use of remifentanil (for analgesia) and midazolam (for sedation) likely masked symptoms of statin-associated muscle syndrome, such pain or weakness. This pharmacodynamic interference explains the absence of reported statin-associated muscle syndrome despite ongoing statin therapy. On admission, the patient’s markedly elevated CK level (6624.80 U/L) and cTnI exceeding 100 μg/L initially supported a diagnosis of ACS. This biomarker profile likely led clinicians to attribute the subsequent rise in CK to ACS progression, inadvertently delaying recognition of the true cause: statin-induced RM. Moreover, statin-associated muscle syndrome typically develops weeks to months after treatment initiation.^[12]^ In this case, the patient had been on atorvastatin for only 3 days – an atypically short onset period. This unusual timeline, combined with diagnostic confounding, contributed to the delay in statin discontinuation. By March 10, the patient’s condition had deteriorated significantly: MYO exceeded 1200 μg/L, left ventricular ejection fraction decreased to 39%, and CRE rose to 792 μmol/L. A diagnosis of ARF complicating heart failure was established. Treatment was initiated with intravenous fluids (5% glucose injection at 100 mL/h). With a 24-hour urine output of only 140 mL, indicating anuria, continuous renal replacement therapy was performed from March 11 to 18. Following this intervention, the patient’s CK and creatinine levels decreased.

Current guidelines recommend initiating high-intensity statin therapy as early as possible in all ACS patients without contraindications, regardless of baseline cholesterol levels. However, as discussed, high-potency statins are associated with a significantly increased risk of myopathy and, rarely, RM. This risk–benefit balance is especially urgent given the rapidly rising incidence of ACS in China. Widespread implementation of these guidelines will expose more patients to high-dose statins, meaning even a low absolute risk may result in a substantial number of adverse events. Therefore, it is imperative to complement standard therapy with robust risk stratification, vigilant monitoring, and patient education to mitigate potential harms. Current guidelines also favor ticagrelor over clopidogrel in ACS, making the combination of ticagrelor and statins increasingly common. However, this combination elevates RM risk since ticagrelor increases systemic exposure to certain statins. When combined with other risk factors – such as East Asian ethnicity and elevated baseline CK – the potential for RM is substantially heightened. The goal of low-density lipoprotein cholesterol (LDL-C) reduction in ACS patients is to decrease the risk of cardiovascular mortality, myocardial infarction, stroke, and recurrent ischemic events. This principle applies to all patients with atherosclerotic cardiovascular disease (ASCVD). Clinical outcomes depend primarily on achieving specific LDL-C targets, the magnitude of LDL-C reduction, and the sustained duration of reduction, rather than the specific pharmacologic agent used.^[13]^ High-intensity statin therapy (e.g., atorvastatin 40 mg or rosuvastatin 20 mg daily) can reduce LDL-C by ≥ 50%. Alternatively, a moderate-intensity statin combined with a cholesterol absorption inhibitor such as ezetimibe can achieve a comparable reduction of 50% to 60%.^[14]^ The efficacy of this strategy was demonstrated in the IMPROVE-IT trial, which randomized 18,144 patients within 10 days of an ACS event to simvastatin 40 mg/d plus ezetimibe 10 mg/d or simvastatin 40 mg/d plus placebo. After a median follow-up of approximately 6 years, the ezetimibe group showed a significant 6.4% relative reduction in major adverse cardiovascular events.^[15]^ Moreover, studies in Asian populations with ASCVD suggest that moderate-intensity statin plus ezetimibe may be more effective than high-intensity statin monotherapy, offering better LDL-C goal attainment, improved tolerability, and a promising trend in reducing cardiovascular events.^[16]^

## 5. Conclusion

In ACS patients with multiple risk factors for statin-induced rhabdomyolysis (e.g., East Asian ethnicity, concomitant use of ticagrelor, elevated baseline CK, clinicians should be aware of the potentially increased risk. Although high-intensity statin therapy remains the guideline-recommended standard of care, a strategy utilizing a moderate-intensity statin in combination with ezetimibe may be a rational alternative to achieve LDL-C reduction goals while potentially mitigating the risk of severe muscle toxicity in this specific high-risk subpopulation. Close monitoring for statin-associated muscle syndrome and CK levels is imperative, regardless of the regimen chosen. For patients without muscle symptoms or who are unable to communicate, CK levels exceeding 10-fold the baseline should prompt immediate discontinuation of statins to prevent progression to more severe RM. If RM has already occurred, prompt aggressive intravenous fluid replacement, monitoring of urine output, and alkalization of urine should be initiated to prevent ARF.

## Acknowledgments

We are also grateful to all the researchers, including the physicians, nurses, and technicians, who participated in this study.

## Author contributions

**Supervision:** Ling Zhao.

**Writing – original draft:** Bin Li.

**Writing – review & editing:** Ling Zhao.
